# Comparison of Partial Volume Effects in Arterial and Venous Contrast Curves in CT Brain Perfusion Imaging

**DOI:** 10.1371/journal.pone.0097586

**Published:** 2014-05-23

**Authors:** Alan J. Riordan, Edwin Bennink, Jan Willem Dankbaar, Max A. Viergever, Birgitta K. Velthuis, Ewoud J. Smit, Hugo W. A. M. de Jong

**Affiliations:** Department of Radiology, University Medical Centre, Utrecht, The Netherlands; Max Planck Institute for Human Cognitive and Brain Sciences, Germany

## Abstract

**Purpose:**

In brain CT perfusion (CTP), the arterial contrast bolus is scaled to have the same area under the curve (AUC) as the venous outflow to correct for partial volume effects (PVE). This scaling is based on the assumption that large veins are unaffected by PVE. Measurement of the internal carotid artery (ICA), usually unaffected by PVE due to its large diameter, may avoid the need for partial volume correction. The aims of this work are to examine *i*) the assumptions behind PVE correction and *ii*) the potential of selecting the ICA obviating correction for PVE.

**Methods:**

The AUC of the ICA and sagittal sinus were measured in CTP datasets from 52 patients. The AUCs were determined by *i*) using commercial CTP software based on a Gaussian curve-fitting to the time attenuation curve, and *ii*) by simple integration of the time attenuation curve over a time interval. In addition, frames acquired up to 3 minutes after first bolus passage were used to examine the ratio of arterial and venous enhancement. The impact of selecting the ICA without PVE correction was illustrated by reporting cerebral blood volume (CBV) measurements.

**Results:**

In 49 of 52 patients, the AUC of the ICA was significantly larger than that of the sagittal sinus (p = 0.017). Measured after the first pass bolus, contrast enhancement remained 50% higher in the ICA just after the first pass bolus, and 30% higher 3 minutes later. CBV measurements were significantly lowered when the ICA was used without PVE correction.

**Conclusions:**

Contradicting the assumptions underlying PVE correction, contrast in the ICA was significantly higher than in the sagittal sinus, even 3 minutes after the first pass of the contrast bolus. PVE correction might lead to overestimation of CBV if the CBV is calculated using the AUC of the time attenuation curves.

## Introduction

CT perfusion (CTP) is used in the diagnosis of acute ischemic stroke to non-invasively measure tissue perfusion parameters[1]–[5]. Although relative perfusion parameters such as relative cerebral blood volume (rCBV), obtained by normalization to the contra-lateral hemisphere, are successfully applied in stroke imaging[6]–[8], the absolute CBV is a more accurate measure for delineating the infarct core[9]. Accurate absolute perfusion values would also be desirable to improve the comparability between different CTP modalities and allow for better comparison within large patient groups or databases. The latter allows for establishment of accurate correlations between CTP parameter values and clinical prognostic information.

Calculation of CTP maps requires selection of an arterial input function (AIF) which is typically measured in the middle cerebral artery (MCA) or anterior cerebral artery (ACA). While several methods exist to calculate CBV, including the impulse residue function approach [10], the determination of CBV by comparing the area under the curve (AUC) of the tissue time–attenuation curves to the AUC of a reference curve is the most widely used method and the focus of this study. The AUC of the first pass bolus of the AIF is used to calculate the CBV by taking the ratio of the tissue and AIF AUC's (Eq 1.) after correction for difference in hematocrit[11].
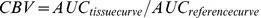
(Eq. 1)


Hence, the AIF should ideally be taken as the reference curve in Eq 1. However, the amplitude of the AIF is commonly underestimated as a result of small-diameter (MCA and ACA) vessels and limited spatial resolution (defined by the in-plane axial image resolution and slice thickness[12]–[15]). Since underestimation of the AUC of the AIF results in overestimation of the CBV (see equation 1), correction for this partial volume effect (PVE) is necessary and typically performed using the venous output function (VOF)[16]–[22]. This correction is based on two assumptions: first, that the centre pixels of a large vein like the superior sagittal sinus (SSS) or the straight sinus, is unaffected by PVE: and second, that without PVE (i.e. an ideal imaging situation), all AIFs and VOF should have equal AUCs. Thus, to perform a correction for the PVE, the VOF is taken as the reference curve in Eq.1. This current clinical method to correct for PVE in the arteries has been necessary for lack of an alternative, and although it is commonly used clinical validations have not been published.

Recent development of CT scanners with larger scan coverage allows selection of the internal carotid artery (ICA) as the AIF reference artery. The large diameter of the ICA, 4.8 mm on average in adult humans[23] and orientation (perpendicular to the slices) potentially allows for direct measurement of the inflow without the need to apply a PVE correction derived from the venous outflow. This is desirable as it reduces potential for user variability, anatomical variations, and measurement errors.

The purpose of this study is to investigate the suitability of selecting the ICA to measure the reference curve for calculation of CBV, and to examine the assumptions behind the clinically used method of PVE correction using the venous time-attenuation curve.

## Materials and Methods

### Patients

An initial group of 80 patients were retrospectively selected from participants in a multicentre stroke trial (the DUtch acute Stroke Trial; (DUST) ClinicalTrials.gov Identifier: NCT00880113). DUST is a prospective study on the prognostic value of CTP and CTA in patients with acute stroke symptoms. The DUST study has been approved by the Medical Ethics Review Committee of the University Medical Centre Utrecht, and all subjects gave written informed consent. Inclusion criteria for this sub-study were as follows: (1) admission scan on a 256-slice scanner (Brilliance iCT, Philips Healthcare, Best, the Netherlands), to ensure a uniform quality of data; (2), CTP with an extended acquisition protocol (detailed below), in thin-slice mode. This initial group of patients was visually assessed by an expert reader to ensure no movement was observable after motion correction, and the ICA was included in the scan coverage. This resulted in 28 patients being discarded and a final patient cohort of 52 patients.

### CTP data

All data were acquired and reconstructed using an extended clinical protocol: 150 mAs/rot, 80 kVp, 512×512 matrix, 200 mm field of view, 0.39×0.39 mm pixels, 81 reconstructed slices with thickness/spacing of 1.0/0.8 mm and a rotation time of 0.33 seconds. The in plane image resolution, measured with an image quality phantom with a 10 µm tungsten wire, was 1.46 mm (FWHM). The geometry of the cone beam with a large field of view meant that the data sets were restricted to 6.5 cm axial coverage. The thin 1 mm slice thickness is required to ensure the absence of PVE in the AIF taken from the ICA should it cross the slice plane at an oblique angle [24], [25]. Forty millilitres of nonionic contrast agent (Iopromide, Ultravist, 300 mg iodine/ml; Schering, Berlin, Germany) was injected into the cubital vein (18-gauge needle) at a rate of 6 ml/s followed by a forty millilitres saline flush at a rate of 6 ml/s by using a dual power injector (Stellant Dual CT injector; Medrad Europe, Beek, the Netherlands). An initial acquisition series of 25 time-frames at 2-second intervals was immediately followed by a second series of acquisitions with six time-frames at 30 second intervals. The initial acquisition constitutes the standard stroke CTP protocol used in our institute, the total CT dose (CTDI-vol) including the extended acquisition was 185 mGy.

### Measurement of arterial and venous AUC's

The AUC of the arterial and venous contrast bolus first-pass was measured at the ICA and SSS. These AUCs were measured using both commercial software employing a Gaussian fit and an alternative method using Trapezoidal Integration (Table 1).

**Table 1:** Outline of AUC measurement methods and analysis.

First pass of contrast bolus		
*AUC method*	*ROI definition*	*Pixel choice*
Gaussian fit (Philips EBW 4.0)	Manual (circle)	Maximum enhancement within ROI
Trapezoidal Integration	Manual (circle)	Center of vessel

After first pass of contrast bolus		
*Method*	*ROI definition*	*Pixel choice*
Temporal ratio of ICA and SSS	Manual (circle)	Center of vessel

### Measurement of AUC using commercial software

**Figure 1:**
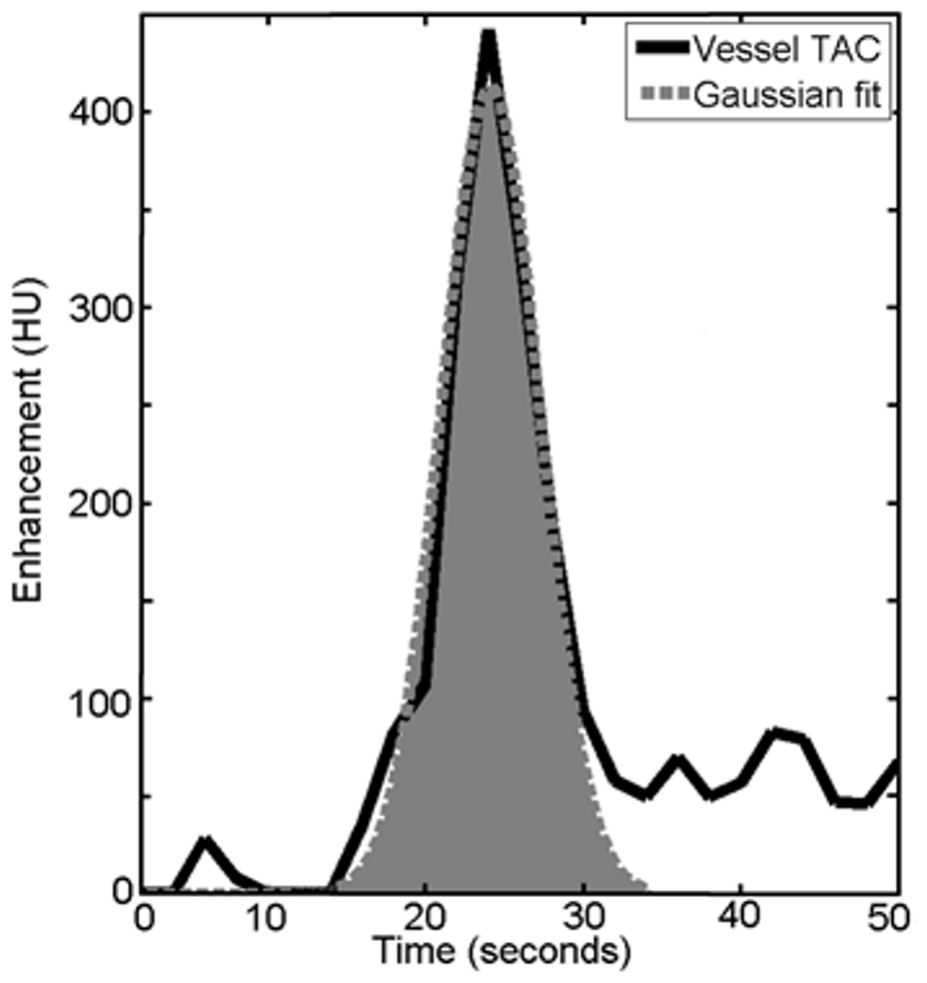
Gaussian fitting of the first pass of the bolus in the ICA time-attenuation curve.

Direct measurement of the AUC of the ICA and SSS was performed using clinical software (EBW 4.0, Brain Perfusion Package, Philips Healthcare, Best, the Netherlands). The ICA and SSS were selected to be approximately perpendicular to the slice plane[19], [24]. Circular ROIs were manually drawn on the blood vessel locations on the maximum intensity projection of the patient data. The software selects the pixel within the circular ROI's with the time-attenuation curve (TAC) that has the maximum enhancement over time. Subsequently, the software estimated the AUCs by fitting a Gaussian curve to the TAC[5]. A Gaussian curve fit distinguishes the firstpass bolus from the recirculation (Figure 1). The AUC of the bolus is therefore defined as the area under the Gaussian shape. This measurement included correction for background, defined as the average HU value before bolus arrival. All post-reconstruction image filtering/smoothing was disabled to minimize any additional averaging of the vessels with the surrounding structures that could influence the partial volume effect. Image registration was performed by the software. For each patient, 4 (+/−1) vessel locations were selected on the ICA in the lower slices at the cavernous sinus portion and ten or more vessel locations were selected in SSS.

### Measurement of AUC analysis using trapezoidal integration

Measuring the TAC at the pixel with maximum enhancement, although common in clinical software, may theoretically introduce a bias towards artificially higher TACs owing to noise. Furthermore, fitting a Gaussian curve to the TACs may introduce an under- or over-estimation to the AUC. To avoid potential bias due to these two factors, an independent set of measurements was carried out on the same vessels without using the commercial software.

Since the vessel centre is the least affected by partial volume from surrounding tissue, the TACs of the vessels were measured at the pixel determined to be at the vessel centre (VC) using a threshold technique applied to the average of all frames of the CTP[26]. Trapezoidal integration of these vessel TACs over the time interval between the bolus arrival and 6 seconds after the peak value was recorded. The bolus arrival time and peak value were determined independently for the arteries and veins, to avoid influence of delayed bolus arrival in the veins on AUC measurement. Prior to this analysis motion correction was applied by a 3D rigid image registration technique[27]. Background correction was also applied by subtracting the average HU value of the frames before arrival of the contrast bolus in the same way as the commercial software.

### Analysis of AUC during first passage of the contrast bolus

**Figure 2:**
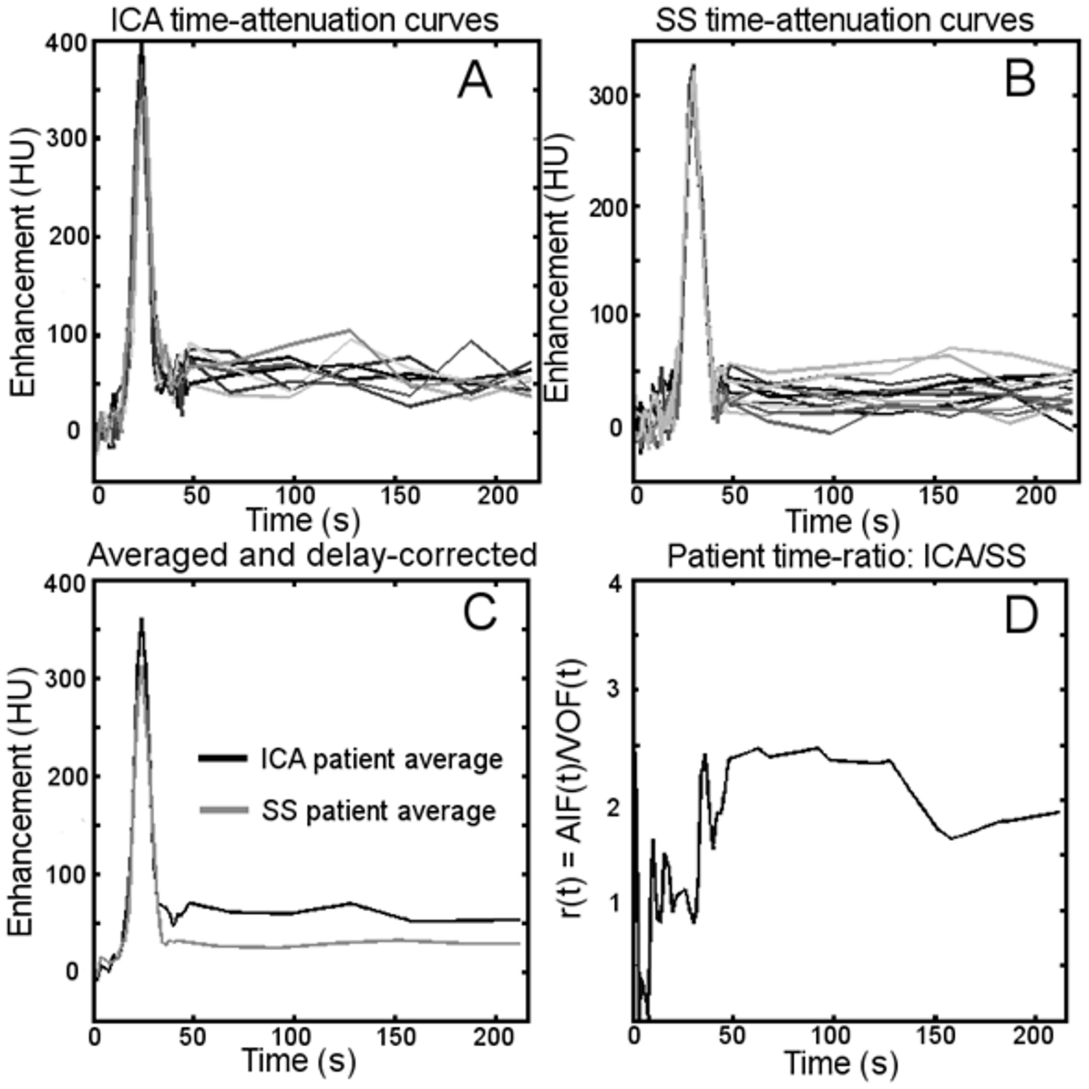
Examples of acquired time-attenuation curves from one patient. The time-attenuation curves were separated into ICA (panel A) and SSS (panel B) measurements. Averaging of these groups gives a typical time-attenuation curve for each vessel, the SSS is then shifted in time to correct for the delay of bolus arrival time in the veins (panel C). The curves are then interpolated to allow a ratio to be calculated in the frames of the extended protocol (panel D).

In order to compare the results between patients, the average AUC measured in the ICA measurements of each patient was divided by the average AUC of the SSS measurements in that patient (Figure 2): *r_AUC_* = AUC_ICA_/AUC_SSS_. This can be considered to be an inter-patient normalization. The ratio *r_AUC_* was independently calculated for the AUCs found with the clinical software and with trapezoidal integration described above.

### Contrast enhancement after first passage of the bolus

The frames from the extended acquisition were used to examine the change in contrast enhancement after the first pass of the bolus (Table 1). The ratio of *r(t) = AIF(t)/VOF(t)* produces a curve that represents how the enhancement in the average of all the ICA (AIF) compares to the average of all the SSS (VOF) throughout the extended acquisition on a single patient. This ratio does not describe the total amount of contrast in the system, merely the relative enhancement over a time period of 3 minutes. This ratio was calculated independently and based on at least 3 arterial TACs and venous TACs for each patient.

For the calculation of r(t), the first the peak enhancement of the TACs were aligned with respect to their timing to correct for the delay of the venous signal relative to the arterial signal. This was done as it was thought that it may be inaccurate to compare a contrast enhancement at a particular time in the vein to that of the artery when the same concentration of contrast had passed though the artery a few seconds earlier. This temporal alignment was accomplished with linear interpolation of the tails so that the 30 second temporal resolution of the extended protocol would not be an issue when comparing time points on the AIF and the time-shifted VOF. A Wilcoxon signed-rank test was used to determine whether there was a statistically significant difference in *r(t)* at the beginning of the extended scan (t = 60 s) and near the end of the scan (t = 180 s). This difference could indicate a trend or convergence towards equilibrium.

### CBV analysis

To illustrate the impact of AUC variations on CBV calculations, on all 52 patients white matter ROI's (6–8 cm^2^) in the frontal lobe were manually drawn and three average tissue CBV's for each patient were calculated according to equation 1, where the AUC(reference curve) was taken from i) the MCA (AIF is MCA with no PVE correction), ii) SSS (AIF is MCA with VOF based PVE correction) and iii) ICA (AIF is ICA with no PVE correction) respectively. All CBV measurements were performed using the Philips EBW 4.0, Brain Perfusion Package Software.

## Results

From the data 52 patients a total of 202 ICA and 674 SSS locations were selected and measured. Examples of ICA and SSS TACs are shown in Figure 2 A/B. In Figure 2C, the SSS curve has been shifted on the time axis to align the peak of the ICA and SSS curves.

### Analysis of AUC during first passage of the contrast bolus

**Figure 3:**
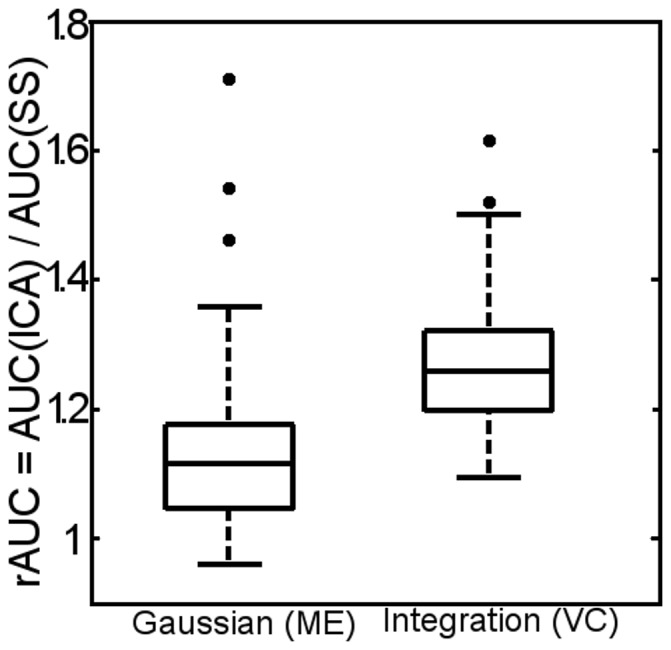
Standard boxplots (with outliers) showing the average ratio rAUC = AUCICA/AUCSSS of all 52 patients. The measurements shown are the AUC determined by the clinical Gaussian fitting at the pixel at the vessel centre (VC) and by integration of the curve at the pixel with maximum enhancement (ME).

Figure 3 shows a standard-type boxplot, with excluded outliers, of *r_AUC_* for all 52 patients. In 49 of the patients the AUC of the ICA was larger than that of the SSS for both measurement methods. The average *r_AUC_* was 1.12 when measured with the clinical software and 1.26 when measured by the simple integration method.

### Contrast enhancement after first passage of the bolus

**Figure 4:**
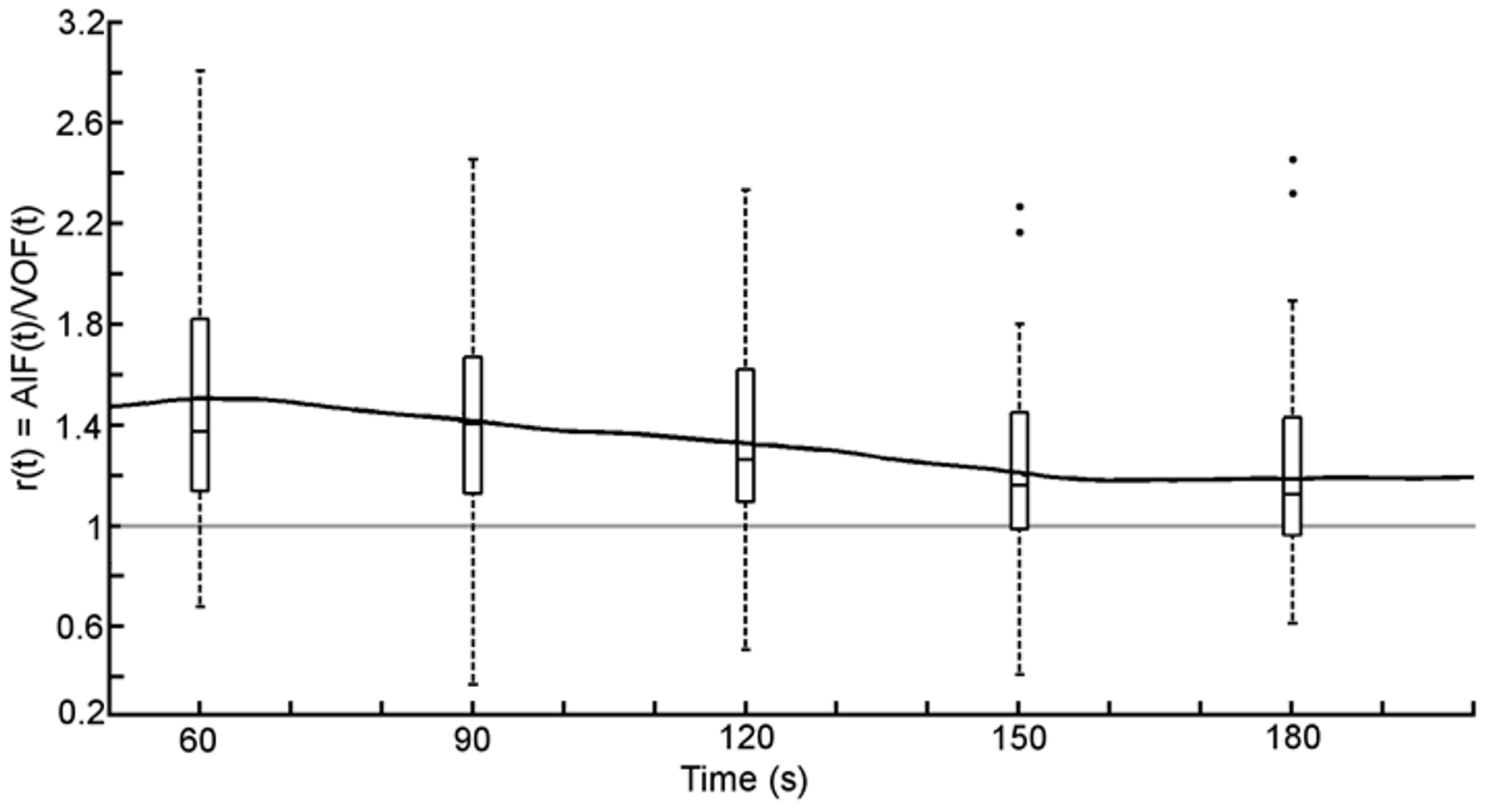
The ratio of the ICA to the SSS over the duration of the extended scan. Each point on the black line is an average of 52 time-ratios. At 30 second intervals a box plot (Solid line is the median value at that time point) representing all 52 patients illustrates the spread of the data across all patients at that point in time.

Figure 4 shows *r_average_(t)* over the duration of the extended scan. This plot is the result of averaging the *r(t)* curves from each of the 52 patients. At 30-second intervals a box plot representing all 52 patients illustrates the spread of the data across all patients at that point in time. The Wilcoxon signed-rank test comparing the distribution of the 52 measurements at 60 s to those at 180 s showed a drop in the mean value of the ratio over 3 minutes to be significant, with a p-value of 0.0014 indicating a significantly decreasing trend.

### CBV analysis

**Figure 5:**
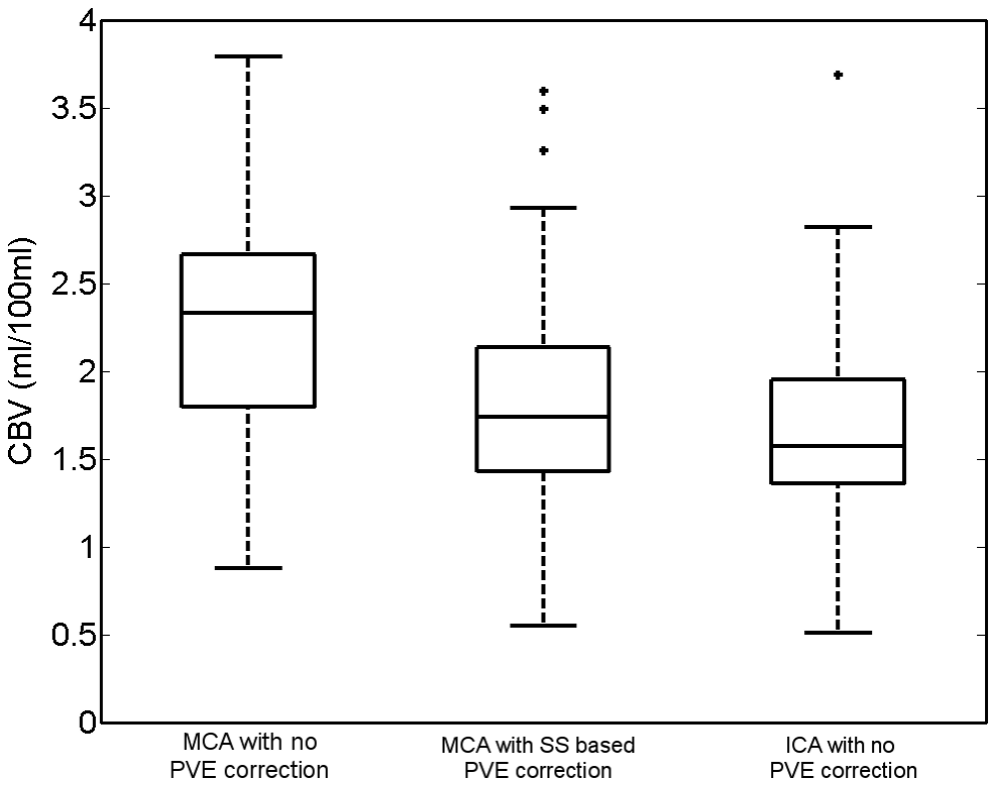
Standard boxplots (with outliers) showing the CBV measurements of ROI drawn on white matter in the 52 patients. Each box represents 52 CBV values calculated from identical ROI but with different vessels selected as the reference vessel. Differences in the CBV between the vessels are therefore directly related to the AUC of the vessel used, and reflect the result found in the direct measurements of the AUC of the vessel time-attenuation curves.

The CBV measurements from each patient, using the MCA, SSS and ICA in turn as the reference vessel are shown in a standard-type boxplot in Figure 5. On average the CBV in white tissue was 2.33 ml(blood)/100 ml(tissue) when calculated using only the MCA, 1.81 ml/100 ml when calculated using the SSS to correct for PVE, and 1.62 ml/100 ml when only the ICA was used without partial volume correction. The differences between the CBV measured in the ICA and VOF corrected measurements were shown to be statistically significant with a Wilcoxon signed-rank test p-value of 0.017.

## Discussion

Modern scanners with large-volume coverage allow for inclusion of the ICA in the CTP scan volume. The large size of the ICA gives the potential for AIF selection without the necessity to correct for partial volume, i.e. without selecting a VOF. This study demonstrates that the ICA is large enough to be unaffected by partial volume on thin slices, implied by the ICA having consistently higher AUC than the VOF in the SSS. This makes PVE correction redundant when calculating CBV from the AUC of the TAC.

Our finding is also contrary to assumptions underlying the clinically applied VOF correction and implies that the ICA is the only viable location to measure the inflow unaffected by PVE when using this clinical protocol and CT scanner. Other scanners may have additional options for AIF selection unaffected by PVE, particularly if they have better image resolution. It is important to recognize from this study that if the VOF is used to correct for PVE it is likely that the CBV will be overestimated.

The CBV measurements illustrate the direct relationship between the AUC measured and the CBV calculated using those AUC. The CBV results using the ICA and SSS reflect the difference found in the more extensive measurement of the AUC of the vessels. The CBV values reported in this study do not represent typical CBV values for healthy brain tissue, the patient group includes patients who have suffered acute stroke of varying severity. No attempt was made to avoid diseased tissue when drawing ROI's to measure the CBV since the only relevant point is how a given ROI returns different values of CBV when the ICA and sagittal sinus are used.

In this study care was taken to avoid biases induced by curve fitting and noise. This was done by measuring the vessels in several independent ways: first, using a curve fit at the pixel with maximum enhancement; second, by simple integration of the curve at the pixel at the centre of the vessel. Additional indications that measurement errors do not underlie our findings can be taken from Figures 2 and 4. These figures present the relative values of the arterial TAC to the venous TAC and show that the venous TAC is lower than the arterial TAC in the frames from the extended protocol. These measurements do not incorporate any curve fitting or integration.

It was observed that at the end of the extended protocol, three minutes after start of contrast administration, the ratio *r_average_(t)* had dropped steadily to a value of 1.3, as compared with a value of 1.5 after one minute. This decline was found to be statistically significant. A similar effect, measuring similar ratios at one and three minutes, was also observed by Lapin et al[28]. With an even longer scan duration Lapin found that it took nearly ten minutes before the enhancement in the arteries and veins was equal, however our clinical protocol does not allow for such a long acquisition period so we are not able to confirm this last finding.

Since a different ratio *r_AUC_* is found with the commercial software and integration method, it is reasonable to assume that one or both of these methods may have a bias due to the measurement method. Methods measuring the AUC of the first pass of the contrast bolus may have bias due to potential sources of error, such as differentiating the first pass from the recirculated contrast or imperfect curve-fitting.

As mentioned in the methods section, trapezoidal integration over a set time period may induce a bias considering the dispersion in the TAC of the vein. However, given that this bias would derive from missing about one second of enhancement on the edge of the shape of the bolus (measured with a temporal resolution of 2 seconds) it is unlikely that it accounts for the large difference (∼25%) between the AUC of the ICA and SSS measured with this method. However we do acknowledge that some of this difference may be due to dispersion.

The third measurement, examining the enhancement after the first pass of the bolus, is an uncomplicated measurement where no assumptions are made in terms of integration or curve fitting. However, all three measurements agree that the average enhancement in the ICA is higher than the SSS, at least 12% higher using the commercial software, and possibly 50% higher when observing the contrast enhancement after the first pass of the bolus.

This last result is the most surprising as it is expected that the contrast, and hence the enhancement, in the entire vascular system should quickly approach an equilibrium after the first passage of the bolus. However, what is observed is that the enhancement in the vessels does not go to an equilibrium for the entire 3 minute duration of the extended scan. The enduring difference in enhancement between artery and vein, observed in all three of the measurements, cannot be explained by calcification in the ICA wall as this is filtered out by the background correction applied by subtracting the average HU value of the first 6 frames before contrast arrival. The difference may be a result of a difference in the hematocrit values between arteries and veins [28], [30]. This hematocrit difference could explain the difference in enhancement observed in the ICA and SSS in the extended frames, while also allowing for the amount of contrast in the entire vascular system to be in equilibrium.

However the possible difference in hematocrit is not sufficient to explain the declining ratio observed in the extended protocol. It has been documented that the high osmolarity of CT contrast agents can acutely increase the blood volume, causing red blood cell crenation[30]. This process may further change the hematocrit value in a way that it is dependent on the size of the red blood cells and the pressure in the vessel, both of which are different in arteries and veins[29]. The hematocrit may thus be dynamic due to the changing concentration of contrast in the blood, thereby explaining our results. However, this explanation is merely a theory, and further study is required to explain why the contrast enhancement remains higher in the ICA long after the system has reached equilibrium.

At present, most commercial CTP software uses thicker slices (3–5 mm) to enhance speed and simplicity of the CTP analysis. These thicker slices may also introduce more partial volume effects[24]. In particular measurements from vessels not perpendicular to the slice plane may suffer from partial volume effects, which will affect the ICA more than the larger venous sinus. This may affect the applicability of our findings to thick-slice CTP data.

One must also realize that the currently used CTP thresholds for defining infarct and penumbra are based on limited-coverage, 5 mm CTP data using the small ACA or MCA as arterial input and venous sinus as compensation for the partial volume effects of these small arteries[9]. Our results show that the AUC of the ICA is higher than the VOF in 94% of the cases on thin-slice data and therefore using the VOF will likely underestimate the arterial inflow and subsequently overestimate the CBV. More accurate CBV results using thin slice ICA as input may therefore require reassessment of thresholds defining the infarct core.

One technical limitation to this study must be acknowledged. Subtle movement of the subject between time frames that cannot be detected visually could affect consistent measurement of TAC, although this can to some extent be corrected for by image registration. This would lower values in the arterial inflow (due to the smaller vessel diameter of the ICA relative to the SSS) more than in the venous outflow, hence finding the AUC of the ICA to be larger than the VOF is in spite of this limitation and not as a result of it.

In conclusion, increased spatial coverage and thin-slice CTP data allows for measurement of the ICA inflow unaffected by partial volume effects. Our results cast doubt on the reliability of the widely used method of partial volume correction that uses the AUC of the venous outflow, since the AUC the large ICA was larger than that of the veins, contrary to the common assumption that both AUCs are equal in the absence of PVE. Selecting a smaller artery, requiring correction for partial volume effect using the VOF, will probably underestimate the AUC of the arterial inflow and therefore overestimate the CBV if calculated using the AUC of the time attenuation curves.
